# Not All Body Surface Area Formulas Are the Same, but Does It Matter?

**DOI:** 10.1200/JGO.2016.005876

**Published:** 2016-07-20

**Authors:** Wasek Faisal, Hoi-Mun Tang, Susan Tiley, Craig Kukard

**Affiliations:** **Wasek Faisal**, Ballarat Regional Integrated Cancer Centre, Ballarat, Victoria; **Hoi-Mun Tang**, Tamworth Hospital, Tamworth; **Susan Tiley** and **Craig Kukard**, Central Coast Cancer Centre, Gosford, New South Wales, Australia

## To the Editor

The method of calculating the chemotherapeutic dose for an individual is still based on a century-old formula for body surface area (BSA), despite its known limitations, mostly due to the lack of any better alternative. In the early days of cancer chemotherapy, anticancer drug doses were either fixed or on the basis of body weight. Prompted by publications by Pinkel^1^ and Freireich et al,^2^ recommending the use of BSA to extrapolate chemotherapy doses from animals to humans, BSA-based dosing started to become common practice in oncology.^3^

The biggest problem with BSA dose individualization is that BSA cannot be measured but has to be estimated with formulas generally incorporating measures of body weight and height. There is no clear evidence for the accuracy of BSA calculations, and the estimation of human BSA has been described as “probably the most difficult of all anthropometric procedures.”^4(p475)^ Currently there are at least five different validated BSA calculation formulas in clinical practice, including Mosteller,^5^ Du Bois and Du Bois,^6^ Haycock et al,^7^ Boyd,^8^ and Gehan and George.^9^ In addition, there is also some controversy regarding the use of actual or ideal body weight for calculating BSA, and it has been recommended that actual weight should be used, including patients who are clinically obese.^10^ Other reports do not support ideal body weight–based empirical dose reductions in obese patients, although calculated doses can be as much as 15% to 30% higher if actual body weight, rather than ideal body weight, is used to determine BSA. In clinical practice, the calculated cytotoxic drug doses are also frequently manipulated by rounding to the nearest convenient dose. Finally, for many patients with cancer, body size will probably vary during the course of the disease because of conditions such as cachexia and anorexia. BSA-based dosing gives the false impression that we are practicing personalized medicine by using a patient-specific metric. This is not entirely true, because BSA does not correlate with other important determinants of drug pharmacokinetics, namely renal and hepatic functions.

We recently attempted to understand how each of the five BSA-calculation formulas perform in an Australian patient cohort, including interformulary variations and whether this would affect day-to-day clinical practice. In our cohort of 224 patients (41% men, 59% women; average age, 62 years; range 20 to 86 years) having chemotherapy for solid tumors in a tertiary cancer center, mean body mass index was 27 (range, 16 to 45). Treatment setting included 112 metastatic (50%), 87 adjuvant (39%), 16 radical-intent chemoradiotherapy (7%), and nine neoadjuvant (4%). The tumor streams covered were 72 breast (32%), 36 lung (17%), 31 colorectal (14%), 16 urologic (7%), 15 pancreas/hepatobiliary (7%), 17 gynecologic (6%), 11 head and neck (5%), nine CNS (4%), six neuroendocrine (3%), seven melanoma (3%), and four others (2%). BSA (mean ± standard deviation) on the basis of each formula were: Mosteller, 1.89 ± 0.21; Haycock et al, 1.91 ± 0.22; Du Bois and Du Bois, 1.87 ± 0.19; Boyd, 1.92 ± 0.22; and Gehan and George, 1.91 ± 0.22. There was good concordance among formulas (*P* = .11). However, when data were filtered to include only patients whose body mass index was > 30 (n = 56), there was significant difference in the calculated BSA among formulas (*P* < .001), with the formulas of Du Bois and Du Bois^6^ and Mosteller^5^ underestimating the BSA compared with the other three. However, this probably would not have a meaningful clinical impact on the actual dose prescribed for the patient because of two observations made by us: first, despite recommendation to the contrary, there is still a tendency to cap BSA at 2.0; and second, the dose-rounding practice (to the nearest 5, 10, or 50 mg, depending on the drug in question) would probably offset the difference in actual dose if a different BSA-calculation formula were used. We tested this hypothesis on our patient population, where we dose calculated for docetaxel, NAB-paclitaxel, capecitabine, irinotecan, fluorouracil, oxaliplatin, and cisplatin using all the different formulas in given patients and found that the rounding effect will eliminate any significant difference in actual dose calculated.

Our study confirmed that, although in an average-built patient any of the validated formulas can predictively and consistently calculate BSA, in an obese patient there is discordance between formulas, which can, in theory, lead to under- or overdosing of cytotoxic drugs. It is important to recognize this limitation of BSA-based dosing and use a single formula in any given institution to minimize dosing variability and maintain consistency. We also noted that despite the recommendations from ASCO and National Comprehensive Cancer Network regarding obese patients, the practice of capping BSA at 2.0 is still quite prevalent. This might be truer in the metastatic setting, where the patients are already quite frail with a poor performance status, and therefore the oncologists are unlikely to be aggressive in their treatment approach, given the palliative intent of the treatment.
